# Development and validation of a predictive model for the risk of developing trichomonas vaginitis in women

**DOI:** 10.1038/s41598-022-24396-y

**Published:** 2022-11-23

**Authors:** Qi Li, Yaqin Li, Ying Bai, Honglei Zhang, Weihong Zhao

**Affiliations:** 1 Department of Obstetrics and Gynecology, the Second Hospital of Shanxi Medical University, Taiyuan, 030001 Shanxi China; 2 Shanxi Medical University, Taiyuan, China; 3 Department of Pathology and Pathophysiology, Basic Medical College, Shanxi Medical University, Taiyuan, China

**Keywords:** Microbiology, Diseases, Health care, Medical research, Risk factors

## Abstract

Trichomonas vaginitis (TV) is the most common non-viral sexually transmitted infection (STI) worldwide. The high prevalence of TV combined with mild or asymptomatic early symptoms leads to clinical vulnerability from delayed diagnosis. Latent infection can increase the incidence of pelvic infections, infertility, and adverse pregnancy complications. Data from 898 women who underwent vaginal flora testing from June 2014 to December 2014 were used to create a nomogram to assess the risk of TV in women in order to guide TV prevention and clinical intervention. The prediction model was evaluated in terms of identification, calibration, and clinical utility using the C-index, calibration plots, decision curve analysis, and internal validation. Predictors in the TV nomogram included age, occupation, yearly income, tea drinking, bathing frequency, menopause, spontaneous abortion, use of contraceptives, history of gynecological surgery, and HPV infection. The C-index of the TV risk prediction model was 0.732 (95% confidence interval: 0.695–0.768). It showed good discriminatory and predictive power. Decision curve analysis indicated that the nomogram had a good net benefit when the threshold probability of TV in women was 2–80%. The established TV prediction model easily, accurately, and quickly predicts the risk of TV onset.

## Introduction

Trichomonas vaginitis (TV) is an extremely common vaginitis caused by *Trichomonas vaginalis* (*T. vaginalis*) and is the most common non-viral sexually transmitted infection (STI) worldwide^1^. The prevalence of TV in women has been reported to be approximately 5.3% globally^2^, but as high as 14.6% among women in nine STI clinics surveyed in the United States^3^, and was reported to be approximately 4.0% in a sample of 40,000 women in Wuhan, China^4^. *T. vaginalis* is a flagellated protozoan parasite that is usually transmitted directly by sexual contact or indirectly through public baths and swimming pools.^5^. Typical symptoms and signs of TV in women include yellowish-green, foul-smelling, frothy vaginal discharge, and itching and erythema of the vulva. However, most patients with TV have mild or asymptomatic symptoms, resulting in latent infections that last months to years^6^. In addition, some patients have a high TV recurrence rate due to resistance of *T. vaginalis* to metronidazole treatment and exposure to untreated sexual partners^7^. Current studies suggest that TV increases the risk of endometritis, adnexitis, infertility, adverse pregnancy outcomes (e.g., premature rupture of membranes, preterm delivery), bacterial vaginosis, cervical HPV infection, and abnormal cervical cytology, as well as acquisition of human immunodeficiency virus (HIV) and STIs^8–11^. Therefore, there are a series of clinical concerns associated with the high incidence of TV, its insidious clinical symptoms, high underdiagnosis rate, high recurrence rate, and the risk of potential complications. There is an urgent need to improve the understanding of high-risk factors associated with the development of TV, which in turn can contribute to successfully guide clinical practice, early diagnosis, and treatment, and reduce the occurrence of complications. Previous studies have investigated on risk factors for TV; however, these consisted of small sample sizes and mostly focused on opportunistic hospital screening, and no predictive models for the risk of TV development have been reported. This study aimed to create a simple, valid and feasible predictive tool to evaluate the risk of developing TV in women based on a large sample of community-based population screening data, that would be of benefit to a larger number of patients.

## Results

### Patient characteristics

In total, 898 patients were enrolled in this study. After the multicollinearity test, the variance inflation factor (VIF) between all 24 factors was obtained to be less than 3, and there was no multicollinearity. Table 1 provides the data of all the participants, including the demographics and diseases of patients in the TV and no-vaginitis groups. The results of the data analysis of the two groups of patients in the population showed that the differences between the two groups were statistically significant (*P* < 0.05) for age, education level, occupation, yearly income, menopause, history of spontaneous abortion, gynecological surgery, and HPV infection, whereas marriage, bathing facilities, bathing method, frequency of changing underwear, menstrual sex, non-menstrual use of pads, hormone replacement therapy, medical abortion, induced abortion, history of vaginitis, history of pelvic inflammatory disease(PID), condom use, and dietary changes were not statistically significant (*P* > 0.05). Women in the TV group were older, had a higher education level, were farmers by occupation, and had a lower yearly income than those in the non-TV group. Women who were menopausal, had a history of spontaneous abortion, or a history of gynecological surgery were more likely to be infected with TV, while women with HPV infection were less likely to be infected with TV compared to women without HPV infection.

**Table 1:** Differences between demographic and clinical characteristics of the TV group and the group without vaginitis.

Characteristics	Total	TV	No TV	*P* value
Participants, n	898	217	681	
**Age, y**				
18–30	31 (3.5)	2 (0.9)	29 (4.3)	< 0.001
31–40	107 (11.9)	12 (5.5)	95 (14.0)	
41–50	294 (32.7)	40 (18.4)	254 (37.3)	
51–60	378 (42.1)	126 (58.1)	252 (37.0)	
> 60	88 (9.8)	37 (17.1)	51 (7.5)	
**Education level**				
Illiterate	44 (4.9)	16 (7.4)	28 (4.1)	0.034
Primary school	154 (17.1)	47 (21.7)	107 (15.7)	
Junior high school	390 (43.4)	79 (36.4)	311 (45.7)	
High school	194 (21.6)	51 (23.5)	143 (21.0)	
Junior college	74 (8.2)	17 (7.8)	57 (8.4)	
University and above	42 (4.7)	7 (3.2)	35 (5.1)	
**Occupation**				
Others	602 (67.0)	130 (59.9)	472 (69.3)	0.01
Farmer	296 (33.0)	87 (40.1)	209 (30.7)	
**Marriage**				
Others	48 (5.3)	13 (6.0)	35 (5.1)	0.627
Married	850 (94.7)	204 (94.0)	646 (94.9)	
**Yearly income, ¥**				
≤ 10,000	324 (36.1)	101 (46.5)	223 (32.7)	0.001
10,000 to ≤ 30,000	404 (45.0)	84 (38.7)	320 (47.0)	
> 30,000	170 (18.9)	32 (14.7)	138 (20.3)	
**Tea drinking**				
Yes	120 (13.4)	34 (15.7)	86 (12.6)	0.252
No	778 (86.8)	183 (84.3)	595 (87.4)	
**Bathing facilities**				
Yes	583 (64.9)	131 (60.4)	452 (66.4)	0.107
No	315 (35.1)	86 (39.6)	229 (33.6)	
**Bathing frequency, times/week**				
≤ 1	560 (62.4)	136 (62.7)	424 (62.3)	0.696
> 1to ≤ 7	333 (37.1)	79 (36.4)	254 (37.3)	
> 7	5 (0.6)	2 (0.9)	3 (0.4)	
**Bathing method**				
Tub	216 (24.1)	61 (28.1)	155 (22.8)	0.108
Shower	682 (75.9)	156 (71.9)	526 (77.2)	
**Frequency_of_changing_underwear, times/week**				
≤ 1	87 (9.7)	24 (11.1)	63 (9.3)	0.63
> 1 to ≤ 7	810 (90.2)	193 (88.9)	617 (90.6)	
> 7	1 (0.1)	0 (0.0)	1 (0.1)	
**Menstrual sex**				
Yes	21 (2.3)	6 (2.8)	15 (2.2)	0.633
No	877 (97.7)	211 (97.2)	666 (97.8)	
**Non menstrual use of pads**				
Yes	88 (9.8)	21 (9.7)	67 (9.8)	0.945
No	810 (90.2)	196 (90.3)	614 (90.2)	
**Menopause**				
Yes	484 (53.9)	164 (75.6)	320 (47.0)	< 0.001
No	414 (46.1)	53 (24.4)	361 (53.0)	
**Hormone replacement therapy**				
Yes	10 (1.1)	4 (1.8)	6 (0.9)	0.239
No	888 (98.9)	213 (98.2)	675 (99.1)	
**Medical abortion**				
No	811 (90.3)	201 (92.6)	610 (89.6)	0.186
Yes	87 (9.7)	16 (7.4)	71 (10.4)	
**Induced abortion**				
No	529 (58.9)	134 (61.8)	395 (58.0)	0.328
Yes	369 (41.1)	83 (38.2)	286 (42.0)	
**Spontaneous abortion**				
No	835 (93.0)	195 (89.9)	640 (94.0)	0.039
Yes	63 (7.0)	22 (10.1)	41 (6.0)	
**History of vaginitis**				
Yes	193 (21.5)	42 (19.4)	151 (22.2)	0.149
No	560 (62.4)	131 (60.4)	429 (63.0)	
Unknown	145 (16.1)	44 (20.3)	101 (14.8)	
**History of PID**				
Yes	60 (6.7)	13 (6.0)	47 (6.9)	0.318
No	674 (75.1)	157 (72.4)	517 (75.9)	
Unknown	164 (18.3)	47 (21.7)	117 (17.2)	
**Use of contraceptives**				
No	833 (92.8)	195 (89.9)	638 (93.7)	0.058
Yes	65 (7.2)	22 (10.1)	43 (6.3)	
**Condom use**				
No	838 (93.3)	208 (95.9)	630 (92.5)	0.086
Yes	60 (6.7)	9 (4.1)	51 (7.5)	
**History of gynecological surgery**				
Yes	207 (23.1)	78 (35.9)	129 (18.9)	< 0.001
No	691 (76.9)	139 (64.1)	552 (81.1)	
**Dietary changes**				
Yes	869 (96.8)	208 (95.9)	661 (97.1)	0.38
No	29 (3.2)	9 (4.1)	20 (2.9)	
**HPV infection**				
Positive	298 (33.2)	47 (21.7)	251 (36.9)	< 0.001
Negative	600 (66.8)	170 (78.3)	430 (63.1)	

*PID* pelvic inflammatory disease.

### Predictor selection

**Figure 1:**
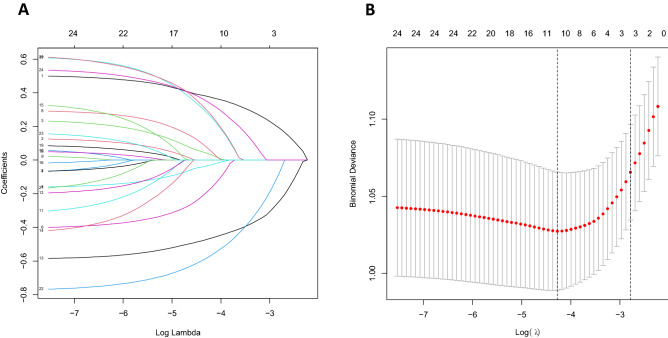
Selection of demographic and clinical characteristics of trichomonas vaginitis using the LASSO logistic regression model. LASSO, least absolute shrinkage and selection operator; *SE* standard error.

Using the LASSO for high-dimensional data reduction, we reduced the 24 predictors to 10 potential predictors (Fig. 1A and B): age, occupation, yearly income, tea drinking, bathing frequency, menopause, spontaneous abortion, use of contraceptives, history of gynecological surgery, and HPV infection.

### Development of personalized prediction models

**Figure 2:**
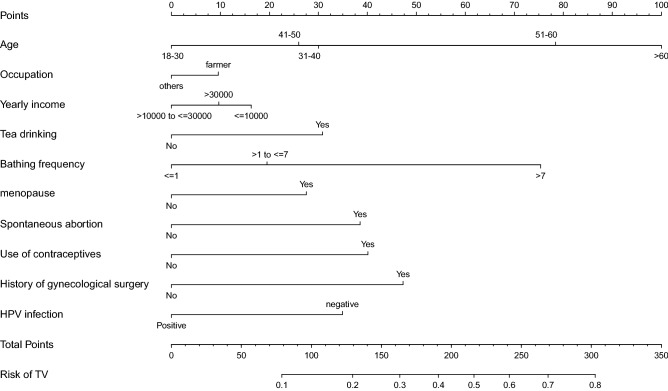
TV risk assessment tool. Points refers to point for the individual risk factor and add together to the total points.

The results of the multiple logistic regression analysis of the 10 predictors mentioned above (Table 2) allowed to develop a model containing these predictors, which was then presented in the form of a nomogram (Fig. 2). The nomogram indicated that increasing age, occupation, yearly income, tea drinking, bathing frequency, menopause, spontaneous abortion, use of contraceptives, and history of gynecological surgery may be risk factors for the occurrence in of TV in women, while HPV infection may be a key protective factor for TV occurrence in women. The line segment corresponding to each variable in the nomogram was marked with a scale that represented the range of values available for that variable. The line segment length reflected the contribution of this factor to the final event. The points in the figure represent the individual scores for each variable under different values. The total points in the figure represent the total score of the individual scores corresponding to the values of all variables. The TV risk in the figure represents the prediction probability of the occurrence of TV, which corresponds to the total of the points.

**Table 2:** Predictors of female TV.

Intercept ant variable	Prediction model		
	β	Odds ratio (95% CI)	*P*-value
Intercept	− 2.640	0.071 (0.011–0.259)	< 0.001
Age	0.457	1.580 (1.208–2.067)	0.001
Occupation	0.145	1.156 (0.805–1.661)	0.433
Yearly income	− 0.106	0.899 (0.695–1.163)	0.418
Tea drinking	0.466	1.594 (1.000–2.539)	0.050
Bathing frequency	0.335	1.397 (0.995–1.963)	0.054
Menopause	0.559	1.749 (1.090–2.807)	0.021
Spontaneous abortion	0.584	1.793 (0.991–3.247)	0.054
Use of contraceptives	0.619	1.858 (1.041–3.314)	0.036
History of gynecological surgery	0.766	2.152 (1.483–3.121)	< 0.001
HPV infection	− 0.539	0.584 (0.397–0.857)	0.006

where β is the regression coefficient. *CI* confidence interval.

### Model performance and validation

**Figure 3:**
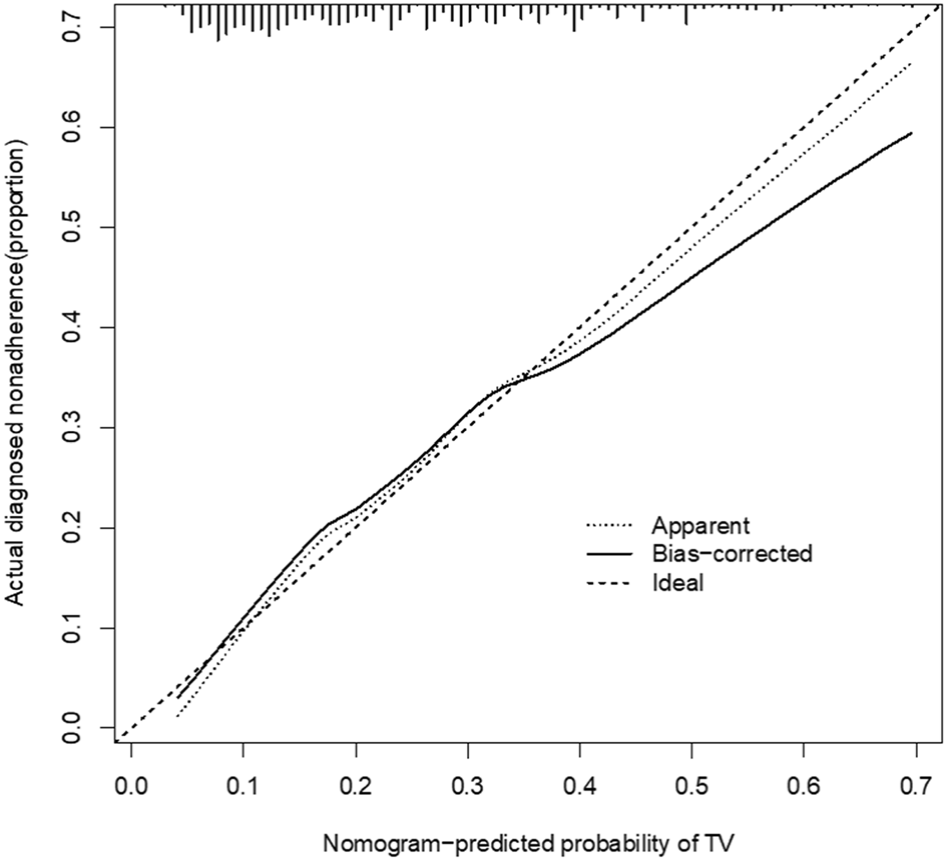
Calibration curves for the ability to predict TV using the nomogram in the cohort.

The calibration curve of the predicted female TV risk nomogram showed good agreement with that of the cohort (Fig. 3). The C-index of the predicted nomogram for this cohort was 0.732 (95% confidence interval: 0.695–0.768) and was confirmed to be 0.707 by bootstrap validation, indicating the good discriminatory power of the model. In the TV risk nomogram, apparent performance demonstrated a good predictive capability.

### Clinical use

**Figure 4:**
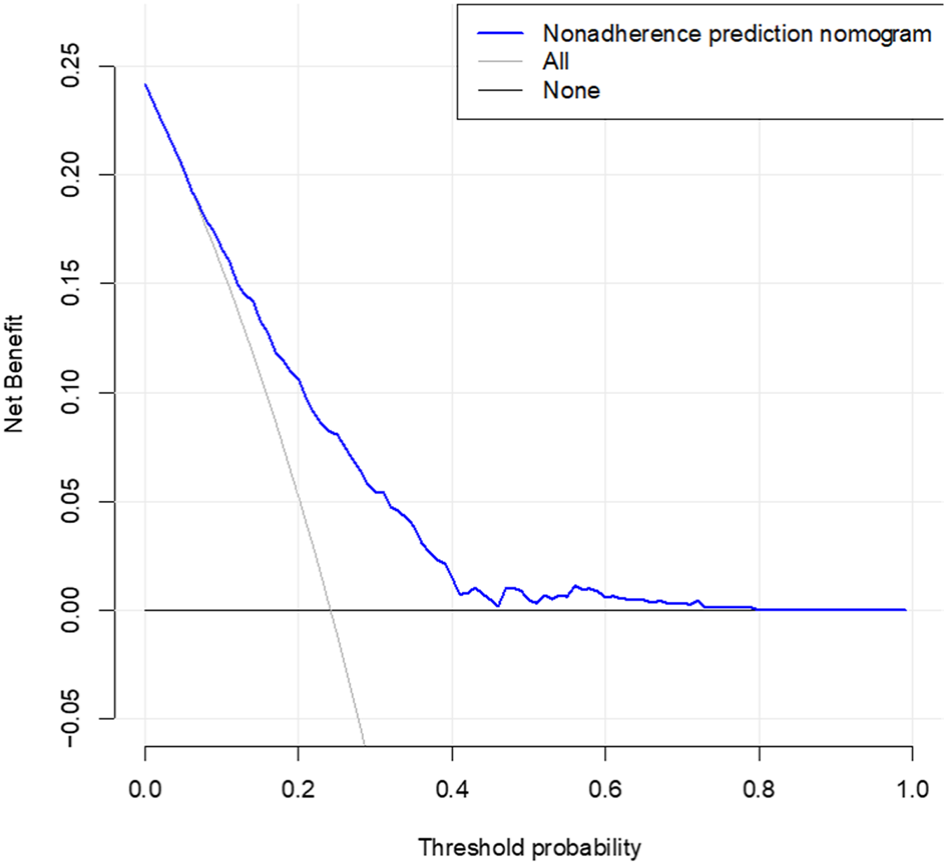
Decision curve analysis of TV nomogram.

This predictive model can be used to assess the risk of TV development in women. By popularizing the model for health care professionals and patients, early and timely anticipatory prevention, diagnosis, and treatment plans can be proposed for women with different risks of TV. The decision curve analysis of the nomogram used in this study is shown in Fig. 4. The decision curve shows that the horizontal and vertical coordinates represent the threshold and net benefit rate, respectively. The blue line represents the clinical diagnostic model for TV. The two black lines represent two extreme cases. That is, the diagonal line represents the assumption that all women had TV, all received the intervention, and the net benefit was an inverse slope with a negative slope; the horizontal line represents the assumption that none of the women had TV, there were no interventions, and the net benefit was zero. When the threshold probabilities of women with TV were > 2% and < 80%, the net benefit of predicting TV using this TV nomogram was higher than that of extreme curves, with a better net benefit.

## Discussion

TV is the most prevalent STI caused by *T. vaginalis* infection and occurs via cross-contamination and sexual transmission. As most patients with TV have mild or no symptoms, early diagnosis and treatment are difficult. Persistence of the infection can cause community transmission and multiple complications, which seriously affect the physical and mental health of women. Therefore, it is important to understand the risk factors for the development of TV to develop effective prevention and control measures. Available data has shown that common risk factors for the development of TV are infected partners, poverty, marital status, older age, and poor hygiene^7^. However, most reports are case–control studies derived from outpatients with symptoms, which are not uniform in either residential background or diet and lifestyle habits, with poor representativeness, which make finding a consensus difficult. The results of the present study will allow for the early diagnosis of many asymptomatic TV patients through large-scale cervical screening, thus shifting the window of TV intervention forward and hopefully radically reducing its incidence. Treating only clinically symptomatic TV patients is highly likely to result in missed diagnoses in asymptomatic patients, leading to a cycle of latent infection and reinfection with *T. vaginalis*, which is clearly not the best option for controlling the occurrence of TV. In this study, we developed and validated a nomogram for predicting TV using ten variables including age, occupation, yearly income, tea drinking, bathing frequency, menopause, spontaneous abortion, use of contraceptives, history of gynecological surgery, and HPV infection to predict the risk of developing TV in women. This prediction model enables personalized risk assessment using variables that are easy to obtain and assess, which is feasible for healthcare professionals and patients, to achieve a rapid assessment for the early detection of TV and allow effective intervention and prevention of long-term complications with good economic and social benefits.

Many studies have shown that increasing age and menopause are strong risk factors for the development of TV in women. Sutton et al. found that increasing age, lower education, and poverty led to an increased prevalence of TV in women of childbearing age in the United States^12^. Stemmer et al. showed that TV did not decrease with age in women and had a higher detection rate in perimenopausal women^13^. The present study also found that increasing age and menopause are risk factors for the development of TV. The reason for this is that the vagina becomes thin and atrophic owing to the lack of hormones in women after age and menopause and is easily traumatized, resulting in serous exudation from the vagina. *T. vaginalis* is prone to survival under such environmental conditions^14^. Simultaneously, the body's defenses against *T. vaginalis* gradually weaken as the immune system declines with age. *T. vaginalis* prefers a higher vaginal pH than normal^15^. After menopause, ovarian function, and estrogen secretion decrease, which increases vaginal pH and susceptibility to *T. vaginalis*.

In the present study, women with occupations such as farming and low annual income levels were more likely to experience TV. Low annual incomes and poverty can affect personal hygiene practices. Farmers are more likely to perform open defecation due to the nature of their work, as well as the poor environment of rural toilets with a large population of users, making them prone to cross-infection^16^. Das et al. also found that people living in poverty were more prone to vaginal infections^17^. Women with low incomes or those living in remote areas of China don’t know about the dangers of TV because of uneven distribution of medical resources and unbalanced urban and rural development. The government should increase the awareness of TV among these people.

The nomogram shows that the frequency of bathing has a great influence on TV infections in women. Good hygiene practices have a positive impact on the balance of vaginal microecology and can reduce the risk of TV infections^18^. Most women believe that thorough bathing must include flushing the vulva and vagina, which can disrupt the balance of vaginal microecology^19^. Too frequent bathing can overclean the vagina and thus alter the vaginal microflora^20^ or predispose to cross-infection, causing invasion of *T. vaginalis* and eventually TV infection.

In addition, we found that tea consumption by women may be an important risk factor for TV. This may be due to the fact that tea contains caffeine, which can easily have a disruptive effect on sleep when consumed improperly^21^; tea also dilutes gastric juices affecting digestion, leading to inadequate nutrient intake in the body^22^; and tea also affects iron absorption, leading to iron deficiency anemia^23^. All these factors can lead to a decrease in human immunity and increase the likelihood of *T. vaginalis* infection. However, some studies have also shown that certain chemicals in tea can have a preventive effect on TV^24^. There is a lack of research on the correlation between tea consumption and TV and should be explored further.

A previous study found a negative association between contraceptive use and *T. vaginalis* infection^25^ and also showed that women who use oral contraceptives are 2.7 times more likely to be infected with *T. vaginalis* than women who do not use the pill^26^. Our study also found that women using oral contraceptives may have an increased likelihood of TV. The use of contraceptives can predispose women to pathogens by altering their immunity and disrupting the vaginal flora^27^. Changes in hormone levels following contraceptive use may result in vaginal mucosal congestion and hypertrophy, as well as vaginal bleeding and pH changes, which favor the growth of *T. vaginalis* in the vagina. The use of oral contraceptive increases the likelihood of sexually transmitted infections, given the decreased reliance on the use of condoms^28^. We recommend the correct use of condoms for contraception, which can be a more preventable measure against TV.

The current study showed that a history of spontaneous abortion and gynecological surgery in women is a strong risk factor for the occurrence of TV. Vaginal bleeding can be caused by spontaneous abortion^29^. After gynecological surgery, women may have decreased immunity due to primary illness and excessive blood loss or post-operative antibiotics that inhibit the growth of lactobacilli, which can disrupt the vaginal micro-ecological balance^30^. In addition, gynecological surgery is likely to alter the vaginal anatomy, which causes abnormal vaginal microflora^31,32^. These factors can lead to susceptibility to TV in women. Clinicians should pay close attention to the postoperative management and follow-up of surgical patients to reduce the incidence of TV.

The nomogram developed in this study indicates that HPV infection is a key protective factor against *T. vaginalis* infection. Although it has been shown that TV affects the ability of vaginal immunity to clear HPV infection by disrupting the vaginal microecological balance^33^, the mechanism by which HPV infection affects TV remains unknown. TV produces symptoms mainly in the cervical and vaginal mucosae. After HPV infection, only a small proportion of patients experience HPV infection, which subsequently develops into cervical cancer. Most patients clear HPV infection via their own immunity within a certain period of time^34^. Therefore, HPV infection may activate the body's immune response, and various immune cells and factors exert defensive functions^35^, thus making the body locally immune and less susceptible to TV.

Older age, menopause, history of spontaneous abortion, and history of gynaecological surgery were independent risk factors and HPV infection was an independent protective factor in this study. Tea drinking, use of contraceptives and bathing too frequency were risk factors. Occupation as a farmer and yearly income had less effect on TV. By combining these ten predictors, a model was constructed to predict model for predicting the occurrence of TV, which is suitable for use in TV clinical risk assessment studies.

The greatest advantage of this study is that all the data derived from a large sample population (40,000 cases) of individuals who underwent mass cervical cancer screening in the same area. Second, the comprehensive questionnaire was designed by epidemiologists and survey factors included a total of 24 indicators across four major components: demographic characteristics, hygiene habits, information on marriage and childbirth, and past medical history, and includes some previously unstudied factors such as dietary changes.

There were several limitations to our study. Firstly, the population in this study was derived from a population with abnormal cervical cytology and external evaluation of more women, especially in multiple centres, is needed for model promotion; secondly, there were some risk factors for TV that were not studied, such as multiple sexual partners; Thirdly, Some of the insignificant variables included in our TV prediction model had a small impact on the model and thus require more future research to confirm and finally, most of the evidence in this study was from cross-sectional studies and it was not possible to assess whether infection occurred after each predictor. Future results from prospective studies are needed to validate and improve the nomogram.

## Methods

### Patient study populationQuery

**Figure 5:**
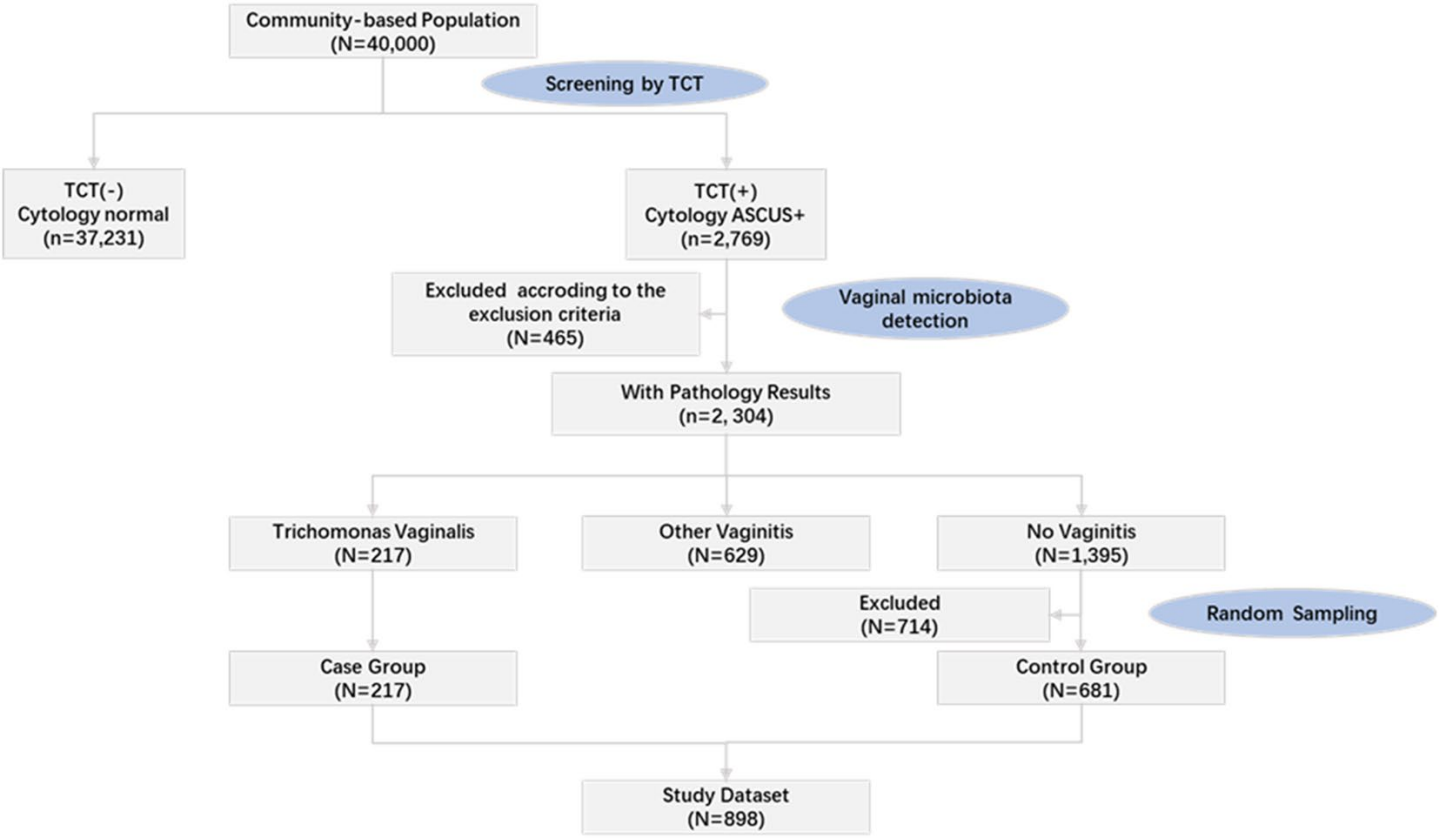
Flowchart of the study participants. TCT: thinprep cytologic test; ASCUS +: atypical squamous cells of undetermined significance or worse.

We analyzed data from 898 women who underwent vaginal flora testing in the Shanxi CIN cohort study from June 2014 to December 2014 (Fig. 5). The procedure was as follows: 40,000 women in Yangqu and Jiexiu areas of Shanxi Province underwent Thinprep cytologic test (TCT), and a total of 2769 cases with atypical squamous cells of undetermined significance or worse (ASC-US) and above were detected. 465 patients who did not meet the criteria were excluded. The exclusion criteria were as follows: (i) 10 cases with abnormal cervical glandular epithelial cells, (ii) 68 patients who refused colposcopy, and (iii) 387 cases with incomplete information (including incomplete information on any of the questionnaires, vaginal flora test, HPV typing test, colposcopic biopsy, and histopathological examination). There were 2304 patients who met the criteria, of whom 217 were diagnosed with trichomoniasis as the case group and 1365 without vaginitis in a random sample of 681 patients as the control group. Patients are not pregnant or menstruating and were not receiving metronidazole (or other) treatment during their menstrual period at the time of the study.

**Figure 6:**
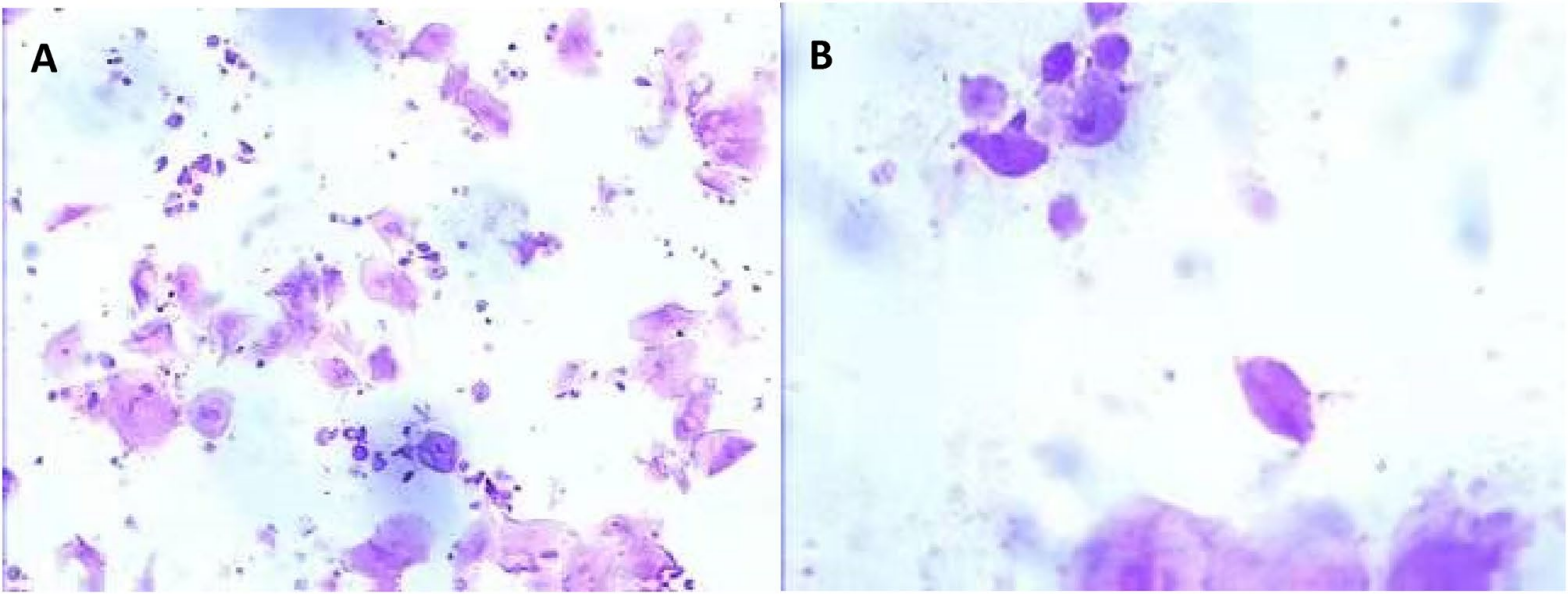
*Trichomonas vaginalis* shown under a 100 × microscope and 400 × microscope.

All the patients who met our inclusion criteria during the study period were included. All examinations and tests were performed under double-blinded conditions. The methods were carried out in accordance with the approved guidelines and regulations. The study was approved by the Ethics Committee of the Second Hospital of Shanxi Medical University. [Ethics Committee Approval Number: (2013) No. (002)]. All participating patients provided written informed consent, completed a uniformly structured questionnaire, and were interviewed face-to-face by a trained and qualified investigator. The survey consisted of four areas: demographic characteristics (age, education, occupation, annual income, and tea drinking), hygiene habits (bathing method, bathing frequency, frequency of changing underwear), marital and fertility information (marital history, sexual history, and contraceptive history), and past medical history (gynecological history, surgical history). Vaginal flora was directly assessed using a sterile cotton swab to obtain vaginal secretions from the upper one-third of the lateral wall of the vagina, which was first used to smear and then examined microscopically for the presence of trichomonads in the secretions (Fig. 6A and B). A dry chemoenzymatic-based combined Vaginitis Test Kit (Jiangsu Shoshi Biotechnology Co., Ltd.) was used as an auxiliary test, which indirectly detected the presence of TV if positive for acetylamino glucosidase and a pH ≥ 4.8.

### Statistical analysis

Statistical analyses were performed using SPSS (v.20) and R (v. 4.1.2) software. A random sample of 681 cases in the vaginal flora test without vaginitis was selected using SPSS software. The chi-squared test (χ2) was used to analyze the relationship between TV and the 24 investigated factors. In R software, the least absolute shrinkage and selection operator (LASSO) was first used to determine the best predictors of TV. Subsequently, using multivariate logistic regression analysis and combining the selected predictors, a prediction model was built, and a nomogram was constructed. These predictors included the dominance ratio (OR) and p-value with a 95% confidence interval (CI). All statistical significance tests were two-sided. Calibration curves were plotted to evaluate the calibration power of the nomogram for the risk of TV. Harrell's C-index was used to assess the discriminatory performance of the TV nomogram. Typically, a C-index greater than 0.7 indicates a reasonable estimate. The relative corrected C-index was calculated using bootstrap validation (1000 bootstrap resamples). Decision curve analysis was performed to assess the clinical benefit of the TV nomogram by quantifying the net benefit of different threshold probabilities.

### Ethics approval and consent to participate

The study was approved by the Ethics Committee of the Second Hospital of Shanxi Medical University. All participating patients provided written informed consent.

## Data Availability

The data used in the current study are available from the corresponding author upon reasonable request.
